# Levels and profiles of pentabromodiphenyl ether contaminants in human breast milk from Riyadh, Saudi Arabia

**DOI:** 10.15537/smj.2023.44.5.20220902

**Published:** 2023-05

**Authors:** Sobhy M. Yakout, Ahmed M. Isa, Amel A. El-Sayed, Ibrahim M. Aziz

**Affiliations:** *From the Department of Biochemistry (Yakout), College of Science; from the Department of Obstetrics and Gynecology (Isa, El-Sayed), College of Medicine; from the Department of Botany and Microbiology (Aziz), College of Science, King Saud University, Riyadh, Kingdom of Saudi Arabia*.

**Keywords:** PBDEs, breast milk, estimated dietary intakes

## Abstract

**Objectives:**

To investigate levels of pentabromodiphenyl ether (PBDEs) in breast milk samples from healthy mothers who had lived in Riyadh for the last 5 years.

**Methods:**

In this cross sectional study, 75 samples were collected and were extracted, cleaned by solid-phase extraction (SPE) and PBDEs analysis was done using gas chromatography–mass spectrometry.

**Results:**

Total PBDEs (∑PBDEs) ranged from 0.2 to 3.6 ng/g lipid weight (lw). BDE-47, -153, -99, and -209 were the dominant congeners. The mothers in this study consumed more meat (69%), followed by the egg (50%), and milk (36%). The majority of donors consumed fish (44%) and egg (33%) 2 times per week. The majority of the participating mothers had completed higher education (68%). All PBDE congeners were detected in the human breast milk samples with high detection frequency (98%). The dominant congener was BDE 47, accounting more than 39% of all BDE congeners, followed by BDE-99 and BDEs 153 which accounted for18% and 12% of the total BDE congeners respectively.

**Conclusion:**

Higher rates of meat and poultry consumption were positively associated with higher breast milk levels of ∑PBDEs. The significant levels of PBDEs that occur in the meat and poultry reared in Saudi Arabia need further investigation especially as Saudis among largest consumers of poultry meat.

**H**uman milk is the best food for babies because it meets all of their nutritional needs and has benefits for their immune systems, mental health, and wallets.^1,2^ It is a unique biological matrix for monitoring certain environmental pollutants since it can offer exposure information for both the mother and breastfed newborn using a non-invasive collecting approach.^3^ Scientists are concerned regarding persistent organic pollutants (POPs) their source and the relationship between exposure, and adverse health outcomes. One of the outcomes of the nation’s efforts is the Stockholm Convention on POPs in 2001.^4^ The Kingdom of Saudi Arabia signed the Stockholm Agreement in 2002 and ratified it in 2012.^5^

Polybrominated diphenyl ethers (BDEs) are a type of POP that exhibits toxicity, resistance to degradation, and bioaccumulation and linked to a variety of health problems.^6-10^ Polybrominated diphenyl ethers are commercially produced in 3 forms: pentaBDE, octaBDE, and decaBDE.^11^ Recent years have seen a dramatic increase in global demand for BDEs due to their excellent flame-retardant qualities, superior thermal stability, and relatively low price.^12^

Since the 1970s, PentaBDE (PBDE) has been used as a flame retardant in polyurethane foam (PUF) and it has minor uses in phenolic resins.^13^ From 1991 to 1999, its global industrial demand went from 4000 tons per year to 8500 tons per year.^14^ In 2007, “there should be no current production of commercial PBDE in Europe, Japan, Canada, Australia, and the United Sates; however, it is possible that production is still going on in the world.^15^

Both inhalation and ingestion of PBDE can lead to its accumulation in the body, stored primarily in body fat and may stay in the body for years.^16^ The PBDE had biomagnification factors in humans 98 higher than any other industrial chemicals studied.^17^ Commercial PBDE mixture, as well as individual congeners, has been shown to cause neurotoxicity developmental, reproductive toxicity and disrupt endocrine function in rodent.^13^

Usage of the PBDE formulation has been banned in many countries worldwide since 2004.^18^ These restrictions will not eliminate PBDE releases from products currently in-service or new products manufactured with recycled PBDE-containing material. The continued use or recycling of consumer products containing PBDEs may be exposing mothers to the chemicals.^19^ This study aimed to investigate levels of PBDEs ether in breast milk samples from healthy mothers who had lived in Riyadh for the last 5 years.

## Methods

In this cross sectional study, 75 mothers, with age range 24-41 years, were fully informed with the nature and objectives of the study and signed a consent form. Inclusion criteria were: lactating mothers with good health and stayed at their current address for at least 5 years prior to sample collection. Exclusion criteria were: mothers with health problems and those who were not willing to participate. The mothers were given a self-reported questionnaire to fill out, which asked for details such as their ages, heights, weights, diets, addresses, dates of birth, and total number of children. The questionnaire is based on the Fourth WHO-Coordinated Survey of Human Milk for Persistent Organic Pollutants, conducted in cooperation with UNEP-Guidelines.^20^ Questionnaire food section has detailed questions on consumption frequencies of fish, dairy products, meat/meat products, and egg/egg products. In line with the Declaration of Helsinki, this cross-sectional study was carried out. The institutional review board (IRB) at King Khalid University Hospital, Riyadh gave their approval (ref, No. 21/0044/IRB) to the study’s protocol, consent form, and questionnaire.

To avoid infection, the mothers requested to wash their hands and breasts using fresh water (without soap or other perfumed detergent) and requested to use single-use gloves before collecting the samples. Approximately 50 mL of milk was got from each mother in a precleaned (solvent-washed) glass container with a Teflon-lined cap, using either a breast pump or by manual expression between the 3 and 8 weeks postpartum. The samples will be immediately frozen and kept at -80°C prior to extraction and analysis.

By following a validated method that included parameters such as limit of detection, and quantification, linearity, reproducibility and precision, we were able to obtain recovery values ranging from 96.5% to 120%. Breast milk samples were shaken for 5 minutes (min) with 10 mL of acetonitrile to extract PBDEs, then vortexed for 2 min and centrifuged for 6 min. Supernatant was transferred to a 2 mL QuEChERS C-18 SPE tube and purified through a 0.2 μm syringe filter directly into a 1.8 ml umber GC vial. The PBDEs were analyzed using triple quadrupole gas chromatography tandem mass spectrometric detection (GC-MS/MS, Thermo Scientific TMQ 8000TM) equipped with GC Column DB-5MS 30m, 0.25mm. The signals in the mass chromatograms and comparisons with internal standards are used to figure out how much of a compound there is. The GC analysis of the cleaned-up extracts were performed in triplicate to evaluate the analytical precision. Throughout the analysis process, standard quality assurance procedures were maintained to ensure accuracy and reliability of the results.

The number of participants required to achieve the desired level of confidence was determined using Raosoft online. To attain a 95% confidence level with a margin of error of 3.15%, a minimum sample size of 75 was calculated to meet the study’s objectives.

### Statistical analysis

The PBDE concentrations were reported based on lipid-normalized values. The residual values in the samples under the detection limits treated as zero to calculate the total PBDE levels. To decrease the uncertainty of the results, only PBDE compounds with >50% of the concentration values above the Method Quantitation Limit (MQL) were included in the statistical analysis. All of the statistical analyses were performed using IBM SPSS Statistics for Windows version 18.0 (IBMCorp, Armonk, NY, USA). A p-value level <0.05 was chosen to indicate statistical significance. Spearman’s rank correlation was employed to investigate the possible associations between the PBDE concentrations and the mother’s age, body mass index, residing time and food consumption.

## Results

### Maternal characteristics

Information such as age, number of births, occupation, smoking, dietary habits, and so forth was recorded for all mothers in (Table 1) as number and percentage of participants or mean with minimum–maximum range. The mean age of this study population was 31.4±4.4 year with age range 24-41 years. Most of participants living in Urban area (98%). Urban populations may be more at risk to be exposed to flame retardants, either at home or in occupational settings, resulting in higher PBDE concentrations. The mean Body Mass Index (BMI) was 25.6±3.0kg cm^2^. Fifty-six percentage of the donor mothers were overweight (BMI, 25–29.9 kg/m^2^) whereas about 7% of them were obese (BMI 30 kg/m^2^ or higher). The PBDEs appear at higher concentrations in fat-containing foods, including fish, meat, eggs and milk. The PBDEs are also present in the human body and traces can be found in human milk. As shown in Table 2, mothers in this study consumed more meat (69%), followed by egg (50%) and milk (36%). The majority of donors consumed fish (44%) and egg (33%) 2 times per week. The majority of the participating mothers had completed higher education (68%).

**Table 1:** - Participants characteristics (N=75).

Characteristic	n (%)
Mean age in years, Mean±SD	31.4±4.4
***Age, years***	
21–25	6 (8.0)
26–30	27 (36.0)
>30	42 (56.0)
Mean BMI, kg/m^2^, Mean±SD	25.6±3.0
***BMI, kg/m** ^2^*	
<25	27 (37.0)
25-29.9	41 (56.2)
30-34.9	3 (4.1)
35-39.9	2 (2.7)
Urban residence	74 (98.0)
***Residence in years***	
≤5	1 (1.3)
>5	74 (98.7)
Rural residence	1 (1.3)
***Residence in years***	
≤5	-
>5	1 (100.0)
***Education***	
≤High school	24 (32.0)
College	8 (10.7)
≥Bachelor’s degree	43 (57.3)
***Birth order***	
First	68 (90.7)
Second or higher	7 (9.3)
***Parity***	
Primiparous	68 (90.7)
Multiparous	7 (9.3)
***Diet (before pregnancy)***	
Omnivore	74 (98.7)
Vegetarian	0 (0)

Data presented as mean±SD for continuous variables whereas categorical variables were presented as number and percentages (%). MBI: body mass index, SD: standard deviation

**Table 2:** - Reported weekly food consumption.

Reported weekly food consumption (persons)	Egg	Fish	Meat	Milk
	n (%)			
Never	0 (0.0)	6 (8.0)	0 (0.0)	0 (0.0)
Sometimes	4 (5.6)	16 (21.3)	0 (0.0)	2 (2.7)
Once a week	3 (4.2)	19 (25.3)	1 (1.3)	0 (0.0)
Twice a week	24 (33.3)	33 (44.0)	4 (5.3)	2 (2.7)
More than twice a week, but not every day	38 (52.8)	1 (1.3)	52 (69.3)	27 (36.0)
Every day	3 (4.2)	0 (0.0)	18 (24.0)	44 (58.7)

Data presented as number and percentages (%).

### The PBDEs levels and their congeners profiles

Table 3 presents descriptive statistics including mean (± SD) and median (Q1-Q3) concentration of congeners found in triplicate measurements from the same breast milk sample. Repeated measures showed that there were no differences in the concentration of congeners in the 3 measurements. Among PBDEs, BDE-47, -153, -99, and -209 were the dominant congeners. The PBDEs level were found in range of 0.2-3.6 ng/g lw and ∑PBDEs was 3.6±2.8 ng/g lw. All PBDEs congeners were detected in the human breast milk samples with high detection frequency (98%). The dominant congener was BDE-47, accounting more than 39% of all BDE congeners, followed by followed by BDE-99 which accounted 18% and BDE-153 that accounted 12% of the total BDE congeners.

**Table 3:** - Mean and median concentration of PBDEs.

BDEs	Mean±SD	Median (Q1-Q3)	N <LOD
BDE-47	1.9±1.8	1.5 (0.7 - 2.8)	2 (2.7)
BDE-99	0.9±0.6	0.7 (0.4 - 1.0)	2 (2.7)
BDE-100	0.2±0.1	0.2 (0.2 - 0.3)	2 (2.7)
BDE-153	0.6±0.5	0.5 (0.3 - 0.8)	2 (2.7)
∑PBDEs	3.6±2.8	2.8 (1.7 – 4.5)	2 (2.7)
∑BDEs	4.9±3.3	3.8 (2.9 – 5.5)	2 (2.7)

Data are shown as mean standard deviation (SD) and median (Q1–Q3). LOD: detection limit, PBDE: Polybrominated diphenyl ethers, BDE: Polybrominated diphenyl ethers

## Discussion

In line with number of other studies,in which BDE-47 or BDE-153 was found to be the most common congener in human and animal samples, our study found that BDE-47 was the most common congener.^21-26^ This high level of BDE-47 in human milk may be caused by exposure to commercial PBDE mixtures, which are mostly made up of BDE-47, -153, and -99. The PBDEs are used to make flame retardants in thermoplastics used in electrical appliances and then mothers living indoor for long time may have higher PBDE levels.^27^ The ∑PBDEs concentrations in this study (average=3.6 ng/g lw) are lower those in the UK, Denmark and Finland^28,29^ but exceed those reported in China,^25,26^ Tanzania,^30^ Uganda,^31^ Philippines,^27^ Vietnam, China, Korea, and Japan.^32^

Previous studies have frequently reported an increase in the accumulation of POPs with maternal age for compounds like polychlorinated biphenyls (PCBs), but not for PBDEs.^23,33^ This could be because PBDEs are a more recent addition to consumer products, resulting in higher ongoing exposure compared to other legacy POPs that have been banned for a longer time.^30,33^ It is also possible that younger women have more contact with PBDE-containing products or consume more PBDE-containing diets, leading to a lack of association between PBDE levels and age. Additionally, factors like parity, diet, living habits, and educational level may confound the age correlation.^34^ The study found no significant association between pre-pregnancy BMI and the levels of BDE-47, -99, -100 and -153 congeners. (Table 4). The observed lack of association was consistent with other studies on PBDEs.^30^ We further investigated the possible association between the maternal diet and PBDE levels (Table 4). Consumption of contaminated food, particularly that of animal origin including fish, meat, milk, and eggs, has been shown as a primary route of human exposure to PBDEs.^31^ The present investigation found no statistically significant links between high intakes of fish, milk, and PBDEs. Similar weak and non-significant correlations between human milk BDEs and fish diet were identified.^35-37^ Poor connections may be explained by differences in how people absorb and metabolize the contaminants. The absence of a correlation could also be due to the fact that PBDEs can come from a variety of different places, including other foods and both indoor and outdoor exposure. Previous research^38-40^ indicates that human exposure to PBDEs does not only occur through food consumption.

**Table 4:** - Correlation between BDE and participant characteristics.

BDEs	Age		BMI		Residing years		Fish and fish Products		Meat and poultry		Milk and milk products		Eggs	
	R	*P*-value	R	*P*-value	R	*P*-value	R	*P*-value	R	*P*-value	R	*P*-value	R	*P*-value
BDE-47	-0.03	0.79	0.1	0.43	0.03	0.80	0.09	0.44	-0.21	0.07	-0.04	0.74	-0.39	<0.01*
BDE-99	-0.03	0.82	0.2	0.09	-0.14	0.23	-0.01	0.90	-0.18	0.12	0.06	0.64	-0.07	0.56
BDE-100	0.04	0.76	-0.04	0.71	0.05	0.70	0.12	0.33	-0.01	0.96	0.08	0.50	-0.05	0.66
BDE-153	0.13	0.28	0.15	0.20	-0.11	0.36	0.07	0.55	-0.1	0.41	0.03	0.77	-0.18	0.014*
∑PBDEs	0.02	0.88	0.19	0.11	-0.01	0.94	0.01	0.95	-0.23	0.05*	-0.05	0.66	-0.31	0.01*

Data presented as spearman correlation (R).

^*^ Significant at *p*<0.05, BMI: body mass index, BDE: Polybrominated diphenyl ethers

In our research, we found that greater rates of meat and poultry consumption were linked to increased levels of PBDEs in breast milk (Table 4). Since Saudis are among the world’s highest per capita chicken consumers, this finding suggests that meat and poultry could be a substantial dietary source of PBDEs. These findings corroborate those of other authors who discovered high levels of PBDEs in a wide range of fatty meals derived from animals such meat and poultry.^41^ The scavenging behavior of the birds exposing them to PBDEs-particle bound pollutants, might find their way into mothers by meat and poultry consumption. Previous studies, has shown that food consumption is not the only significant way humans are exposed to PBDEs.^38-40^ Environmental scientists and epidemiologists should also focus on other routes, such as dust, for monitoring PBDE levels. This suggests that the reported PBDEs amounts in breast milk are due in large part to air deposition of the contaminants from point sources. Therefore, it is necessary to conduct further studies on the levels of PBDEs in air, indoor and outdoor dust samples, and other dietary components in Riyadh.

### Study strength and limitations

The study strength includes the homogeneity of the population, providing first-hand evidence of the association of breast milk PBDEs level with maternal age, BMI, and diet in Riyadh. However, the study’s capacity to make generalizations was restricted due to limited of individual participants. Additionally, the study lacked sufficient data on PBDEs in food matrices and did not account for exposure factors like indoor and outdoor dust contamination. Furthermore, the use of non-reliable data collection tool for diet might have led to paradoxical results especially concerning egg consumption as the results indicate that more consumption of eggs is correlated with lower levels of BDEs. Therefore, further investigations with reliable data collection tool for diet are needed.

In conclusion, our study established the initial concentrations of 4 PBDE congeners in breast milk from Saudi Arabia’s capital. BDE-47 was the most predominant congener (contributed 52% to the ∑PBDEs), followed by BDE-99 (25%). The PBDEs appear to be at higher concentrations in fat-containing foods, including fish, meat, eggs and milk then some traces appear in breast milk. Higher rates of meat and poultry consumption were positively associated with higher breast milk levels of ∑PBDEs. The significant levels of PBDEs occur in the meat and poultry in Saudi Arabia needs further investigation especially Saudis are one of world’s largest consumers of chicken
